# Some Remarks on Hernia

**Published:** 1893-11-25

**Authors:** Wheelton Hind

**Affiliations:** Assistant Surgeon, North Staffordshire Infirmary


### The Hospital Clinic.

[*The Editor will be glad to receive offers of co-operation and contributions from members of the profession. All letters should be addressed to* The Editor, The Lodge, Porchester Square, London, W.]

### SOME REMARKS ON HERNIA.

By Wheelton Hind, M.D., B.S., F.R.C.S., Assistant Surgeon, North Staffordshire Infirmary.

There is perhaps no surgical mistake more frequently made in general practice than that of not recognising the symptoms of strangulated hernia, a mistake fraught with the most disastrous results to the unfortunate patient. How often this mistake is made hospital surgeons are perhaps alone aware, and much to their regret the extent to which the mortality tables, after operation for strangulated hernia, are increased by the too tardy recognition of the *causa mali* is not at all creditable to the profession at large.

It is a golden rule in all cases of vomiting to ascertain the condition of the abdominal rings, especially in cases where a hernia has been in existence for some time. The patient, I am aware, often does not recognise the *fons et origo mali*, and would in his account tend to mislead his medical man; but the latter, too, often forgets that it is his duty to reason, from the narrated facts, inductively, and to ascertain by experiment or observation whether his induction be correct or not.

There is no more satisfactory class of case for operation than a hernia, whether it be strangulated, incarcerated, or otherwise, provided always that it comes into the surgeon's hands early, before pressure and constriction have had time to produce secondary changes in the bowel, and before the repeated attempts at taxis by hands too timid to perform a simple operation, but guided by an ostrich-like mind, which fears no evil as long as none is visibly perceptible, have produced severe injuries in the congested gut. A cutting operation is really much less dangerous, and is accompanied with the advantage that the state of the bowel can be observed and the best treatment therefore adopted, and further that in the large majority of cases a radical cure of the condition producing or allowing hernia can be made. The operation of herniotomy is one which itself causes very little shock, and which may safely be performed at any age. I have often heard my colleagues at the North Staffordshire Infirmary say that in their experience operation by the knife for the radical cure of hernia has never been fatal, which means the results of 30 years' practice. In operating for strangulated hernia, of course, the great difficulty is the great variation of the contents and anatomy of the parts. No two cases seem exactly alike, and it is surprising what a number of different conditions may be found, but the experience gained from a few cases is enough to dictate the best method of procedure in others. It is unfortunate, perhaps, that a large number of medical students do not see either the cases or operations for strangulated hernia, as these are always urgent and must be done as soon after arrival at the hospital as possible, often occurring too after the dressers and students have gone home, and unless some one of the higher appointments necessitating residence be held, the amount of actual experience to be obtained is very small. To obviate this, I think students would be wise to take lodgings for some few months at least close to their hospitals so that they could be summoned to urgency operations.

It is not my intention to encroach on text-books to describe the methods of procedure in operating for hernia, for all this is well and fully described by numerous authors, and the only thing wanted over and above this individual experience can never be obtained second hand, and thanks to the large number of hospitals all over the kingdom no one need be without the latter. From the hospital surgeon's standpoint the points I wish to emphasise are

1. The early recognition of strangulated or incarcerated hernia by investigating carefully the cause of the persistent vomiting, which is a more reliable symptom than constipation, as the descent and nipping of the bowel may occur soon after a motion, in which case the absence of history of obstruction would be misleading.

2. Even where there has been no history of hernia, given an inguinal or femoral tumour, this should always be explored if there be persistent vomiting. I have three times seen this done, however, without finding a hernia. In one case of acute and persistent vomiting both abdominal rings were explored, as there was doubtful fulness, but nothing was found; abdominal section was then performed, and the cause, a band constricting the bowel, was removed. In one other case an encysted hydrocele, and another a sarcoma of the cord were found, but although in these three cases no hernia was discovered, still the rule will be found a safe guide in practice.

I was asked to see a maiden lady of 36, who was complaining of vomiting, which occurred daily without any other gastric symptoms, but was accompanied by a dragging pain in the stomach. There was, I was told, a small lump in the right groin, which was not tender or painful, and the bowels were regular. I suggested an exploration, and found adherent omentum, which had been in turn made into a sac, into which I expect at times a small piece of bowel came down. I removed the omentum and sac and ligatured the ring, performing a radical cure, which has been in every way successful.

A somewhat similar case I saw in consultation with Mr. Vernon. A lady had had repeated attacks at long intervals of vomiting and abdominal pain, sometimes accompanied by constipation. There was an elongated irreducible swelling on the left side of a tense character, which was said to become more painful during the attacks, It came down into the labium below, but passed upwards and outwards along Poupart's ligament. It was very tender on touch, so much so that the question of its inflammatory nature was discussed; the bowels were regular, and the tongue fairly clean. I persuaded the patient to allow an exploration, which I performed, and found the mass to consist of omentum adherent below, but pushing its way upwards and outwards in a diverticulum of the main sac, in front of the external oblique. I removed sac and omentum, and the patient has since been free from all symptoms of her old attacks. The reason in these two cases of the absence of constipation and obstruction is due to the non-descent of bowel in the sac, and the absence of urgent symptoms to the fact that omentum is much more tolerant of pressure than gut.

3. I would emphatically declare that taxis, except the very gentlest and for a very short time, is most dangerous. It is often difficult enough to get the bowel back when the sac is opened and the ring exposed, and it must be useless to attempt for any length of time a method of procedure which is capable of producing fatal results by bruising and lacerating the inflamed gut, and which will militate considerably against the success of any subsequent operation. If it only could be generally recognised that the operation by the knife is far less dangerous than taxis, is far more certain and accurate in the result, and can in addition produce a complete cure, less time would be wasted in an often worse than useless treatment.

4. Palliative treatment should never be adopted as the routine practice, though it may be justifiable for a short time to relieve the symptoms by other than operative means. Of these, position, ice and opium are too well known to need comment, but I will mention a case of a lad of six years old, whose bowel came down into an old hernial sac and became strangulated. I saw him in the country while on a round, and having attempted very gentle taxis unsuccessfully, returned home for the necessary instruments, &c. On my return, however, I found that the mother had reduced the bowel by sitting in a rocking chair, with the child inverted with his thighs round her neck while she gently oscillated to and fro, a method which might be adopted again in young children with advantage.

I may say I always look upon a death from strangulated hernia as a reproach to someone; it may be that the friends of the patient are in fault in not calling in skilled help early enough, but I am sorry to say that very often valuable time and opportunities are allowed to pass by, and operative assistance is only called in at the eleventh hour, even when the case is in the hands of duly qualified medical men.

One of my earliest cases was allowed to go unrelieved for four days, the persistent vomiting being put down by the medical attendant to pregnancy, the patient being six months' pregnant, and the tumour in the groin being regarded as syphilitic enlargement of a gland, although it was partly resonant. Fortunately the constriction was not very tight, and no injury had been done to the bowel by the long delay, and the patient made an uninterrupted recovery, delivery subsequently taking place at the normal period.

Dangerous and nearly always fatal as delay is, spontaneous recovery from a strangulated hernia is not unknown, as the following case related to me by my colleague, Mr. Folker, consulting surgeon to the North Staffordshire Infirmary, will show. The patient was an old woman, who was seen for fæcal fistula in the groin, of which she gave the following quaint history: "A lump came in my groin, which after a bit gathered and burst, after the application of poultices, after which the motions came that way." Mr. Folker took the case into the infirmary to perform Dupuytren's operation, but the rest in bed and preparatory treatment were so beneficial that when the operation day came it was found that the fistula was cured.

It is, I think, always advisable except, perhaps, when operating on cases which are in a state of collapse, to attempt to perform a radical cure. To this end I regard it essential to remove or completely disconnect the sac from the peritoneum. The greatest difficulty lies in removing the sac in many cases, especially where the hernia is congenital, owing to the way in which the tissues of the cord are spread out over the sac. In cases of congenital hernia it is necessary to leave the lower part of the sac wherewith to form a tunica vaginalis scrota. I have seen more than once the vas deferens accidentally cut through in separating the sac from the neighbouring tissues, without any subsequent atrophy of the testicle, and this accident is very liable to occur without the greatest care. In fact, some surgeons advocate and perform removal of the testicle and cord as far as the internal ring, asserting that thereby completely closing the inguinal canal, the liability to a return of the hernia is diminished, and the risk of suppuration and atrophy avoided. I consider that the removal of a sound organ, unless for any cogent reasons, is unwarrantable, and consider the argument that an organ should be removed in order to avoid a possible, but I think somewhat rare, complication, is altogether absurd; we might as well amputate legs in early life for fear they might be broken. In performing the operation for radical cure I adopt no formal method. Each case must be judged upon its own merits. I may, or I may not, suture the pillars of the ring, but if the aperture is wide I always do so. I always remove prolapsed omentum even though in a healthy condition, because it is sufficiently long to lie about in the neighbourhood of the internal ring, into which it may pass, and force its way down again into a new sac. As I said above, the one essential, I believe, is to remove or entirely obliterate the sac. The peritoneum is unlikely to be loose enough to form a second after the first has been entirely taken away.

Two accidents after removal of omentum have come under my notice, the latter, however, only in my own practise.

In the first case, that of a man in the prime of life, on opening the sac omentum only was found, which was ligated and cut off; in a day or so, fatal peritonitis supervened, and it was found post-mortem that the omentum had come down into the sac, doubled together, the free edge never leaving the abdomen, and on ligation and removal of the loop the free edge had been left detached within the abdominal cavity and had become gangrenous. Since this sad experience I always make a point of unfolding and carefully examining the portion of omentum I am going to remove.

A more serious accident happened to me early in the year. I was doing a radical cure for a miner, and on cutting down and opening the sac found it contained nothing but omentum. This was thin and vascular, and having ascertained that I had the free edge, I proceeded to transfix and tie in a Staffordshire knot with a silk ligatum. The first ligatum broke as I was tying the second knot, and a second was applied and successfully tied and the omentum cut off. I then noticed that very little omentum seemed contained in the knot, and there seemed free oozing from the canal. While examining to see the source of the hæmorrhage the rest of the omentum slipped up into the abdominal cavity. The oozing became freer, and evidently seemed to come from within the peritoneum, and as there was no way of reaching the bleeding points by the original incision, I opened the abdomen at once in the median line and found it full of blood, which was coming from the cut edges of the omentum, for the ligature had cut through the soft tissues on either side and contained only the centre portions. Several silk ligatures were placed in a row along the free edge of the bleeding omentum, and after much time and trouble all hæmorrhage stopped. The abdomen was then washed out with warm boracic solution and sewn up. The patient recovered without a bad symptom. I am not aware that an accident of this description has been recorded, but it has taught me to be more careful in the cases with thin omentum not to tie too tightly.

Perhaps the greatest trouble in cases of radical cure is with the ligature around the sac. This often is not sufficiently buried in tissue to become organised or absorbed, and a small sinus keeps open and discharging for weeks after the discharge from hospital. I prefer the use of silk, as it is more easily rendered aseptic than gut. Nowadays it is hard to understand why it was considered necessary to refresh the edges of the ring before bringing them together, the very apparent fact that after an incision and dissection they were already raw and in a condition to become surrounded with plastic lymph, was entirely overlooked by some of the most astute observers, whose elaborate mechanical methods have now given way to more simple and rational procedures.

I generally advocate the operation for radical cure of hernia in all cases of the inguinal and femoral forms after about four years of age; before this, only when the hernia is very large, and when it cannot be kept in position by pads, but as long as the patient is in fair health, I do not deem advanced age a drawback to the operation. I have operated very successfully in many cases over seventy where the bowel was strangulated, and was surprised at the small amount of shock caused by the operation. In young adults, the time spent in bed during the radical cure is well spent, and saves hours of discomfort and annoyance, not to speak of the absence of ever-present danger from strangulation, even with the best fitting trusses. Very few of the cases operated upon fail; and when all the lymph thrown out round the ligatures and neck of the sac has become thoroughly organised there is no need to continue to wear a truss. I generally order my patients to wear a truss for from six to nine months after operation, when I think it can be dispensed with.

